# Spatial disparities of HIV prevalence in South Africa. Do sociodemographic, behavioral, and biological factors explain this spatial variability?

**DOI:** 10.3389/fpubh.2022.994277

**Published:** 2022-11-11

**Authors:** Chigozie Louisa J. Ugwu, Jabulani R. Ncayiyana

**Affiliations:** Public Health Medicine, School of Nursing and Public Health, University of KwaZulu-Natal, Durban, South Africa

**Keywords:** spatial disparities, HIV prevalence, GAMs, sociodemographic factors, behavioral factors, biological factors

## Abstract

**Background:**

In 2021, an estimated 38 million people were living with human immunodeficiency virus (HIV) globally, with over two-thirds living in African regions. In South Africa, ~20% of South African adults are living with HIV. Accurate estimation of the risk factors and spatial patterns of HIV risk using individual-level data from a nationally representative sample is invaluable for designing geographically targeted intervention and control programs.

**Methods:**

Data were obtained from the 2016 South Africa Demographic and Health Survey (SDHS16). The study involved all men and women aged 15 years and older, who responded to questions and tested for HIV in the SDHS. Generalized additive models (GAMs) were fitted to our data with a nonparametric bivariate smooth term of spatial location parameters (X and Y coordinates). The GAMs were used to assess the spatial disparities and the potential contribution of sociodemographic, biological, and behavioral factors to the spatial patterns of HIV prevalence in South Africa.

**Results:**

A significantly highest risk of HIV was observed in east coast, central and north-eastern regions. South African men and women who are widowed and divorced had higher odds of HIV as compared to their counterparts. Additionally, men and women who are unemployed had higher odds of HIV as compared to the employed. Surprisingly, the odds of HIV infection among men residing in rural areas were 1.60 times higher (AOR 1.60, 95% CI 1.12, 2.29) as compared to those in urban areas. But men who were circumcised had lower odds of HIV (AOR 0.73, 95% CI 0.52, 0.98), while those who had STI in the last 12 months prior to the survey had higher odds of HIV (AOR 1.76, 95% CI 1.44, 3.68).

**Conclusion:**

Spatial heterogeneity in HIV risk persisted even after covariate adjustment but differed by sex, suggesting that there are plausible unobserved influencing factors contributing to HIV uneven variation. This study's findings could guide geographically targeted public health policy and effective HIV intervention in South Africa.

## Introduction

According to the 2021 epidemic update of the Joint United Nations Program on HIV/AIDS (UNAIDS), an estimated 38.4 million (33.9–43.8 million) people globally were infected with HIV (1). Despite significant progress in HIV treatment, prevention expansion, and scaling-up, 1.5 million (1.1–2.0 million) new HIV infections and ~6,60,000 deaths from HIV/AIDS-related illnesses were estimated in 2021 (1). Sub-Saharan Africa has the world's largest HIV epidemic, accounting for ~70% of the global HIV disease burden with nearly one in every twenty adults (5%) infected yearly (2, 3).

In South Africa, ~8.3 million adults are living with HIV (4, 5). Despite significant progress in the implementation of control and intervention programs over the years, South Africa accounts for 20% of new HIV infections and 20% of all people living with HIV globally (6). The World Health Organization (WHO) recently endorsed the 2022–2030 Global Health Sector Strategy (GHSS) under the United Nations Sustainable Development Goal 3 (UN-SDGs-3) (7), which aims to target HIV control and prevention by reducing the global HIV burden by at least 50% in high-burdening countries. To meet the SDGs and UNAIDS 2030 HIV reduction goal, an effective surveillance system that uses advanced statistical models to understand how HIV spreads across space and to identify important risk factors that vary by location may be invaluable.

Policymakers are frequently interested in the geographical distribution of disease outcomes or their associations with different risk factors, as well as how these factors exert influence in different geographical locations. Mapping the spatial patterns of HIV prevalence has become a critical tool in this regard. Adopting a spatial approach to HIV prevalence analysis in South Africa is critical because it will aid in identifying specific areas with high or low risks, thereby assisting in HIV intervention planning and allocation of scarce resources. Some epidemiological studies on HIV have confirmed a complex relationship between poverty and economic deprivation and HIV prevalence (8–12). In Eastern and Southern Africa, HIV/AIDS researchers also discovered factors associated with HIV infection such as biological susceptibility, transactional sex, sociodemographic factors, age-disparate relationships, lack of quality education, multiple sex partners, widespread poverty, and unemployment (13–18). Others have reported that geographical and demographic factors influence HIV prevalence (19–22). However, little is known about the spatial variation and disparities in HIV prevalence, particularly among South African men and women over the age of 15 years, and the combined effects of behavioral, socioeconomic, and demographic factors on HIV prevalence in this population.

The geographical distribution of HIV varies to a great extent between adjacent regions that share related risk profiles (23, 24), and many epidemiological studies have used spatial analysis to map spatial variability of disease prevalence to delineate the spatial distribution of disease risk, identify important disease risk factors of public health concern, and predict disease responses in different geographical regions (25–28). The spatial analysis method can provide insight into disease prevalence risk by simultaneously adjusting for confounding factors while accounting for the disease's heterogeneous spatial patterns (24). The geographical variation in HIV prevalence in South Africa has not been fully studied with recent national-level data despite being the world's largest HIV epidemic country. Spatial analyses of individual-level data may provide insight into factors that may contribute to HIV prevalence, and death (29). Therefore, it is critical to gain a better understanding of the magnitude and heterogeneity of HIV prevalence in South Africa, and the associated sociodemographic, behavioral, and contextual risk factors.

In this study, we aimed to investigate spatial disparities of HIV prevalence in South Africa. Furthermore, this study sought to investigate the impact of socioeconomic, demographic, behavioral, and geographic factors on underlying spatial disparities of HIV prevalence.

## Methods

### Data

The data utilized for this study were obtained from the SADHS 2016 (30). The survey covered 99% of the South African population and collected information from a nationally representative sample of 11,083 households covering 8,519 women ages 15–49 years and 3,618 men ages 15–59 years. Detailed survey method, sampling approach, and data collection methods are described elsewhere (31). Households were selected through a two-stage sampling procedure, where the enumeration areas (EAs) were randomly selected, and then households within selected EAs were also enumerated and randomly selected for surveying. The enumeration was repeated for each survey such that the entire population would be represented in each survey. The DHS survey did not differentiate between an incident and prevalent cases, women aged 15–49 years and men aged 15–59 years living in the surveyed households were eligible for questionnaire and HIV testing with dried blood spots. The latitude and longitude of each of the enumeration areas were recorded and privacy was maintained by randomly displacing households by up to 2 km for urban households and up to 5 km for rural households, with a random 1% of rural households being displaced by up to 10 km. The coordinates of the EA in which the final displaced household lies were recorded for spatial analysis. For this study, the individual women's recode, men's recode, and HIV files were merged. Overall, a total of 2,485 women and 2,537 men aged ≥ 15 years were included in our analyses, after removing those with incomplete information. Due to differences in the HIV prevalence, we stratified all the analyses by gender. All data analyses were adjusted for unequal sampling probabilities using survey weights as described in the DHS data analysis guideline (31).

### Statistical and spatial analysis

Descriptive statistics were used to assess the socio-economic, demographic, and behavioral correlates of HIV risk factors. Subsequently, we conducted a spatial analysis using the Generalized additive models (GAMs) with locally weighted regression (LOESS) for a two-dimensional predictor (32, 33), to examine the association between geolocation and HIV infection among men and women over the age of 15 years in South Africa. The GAMs were implemented using MapGAM package in R Version 4.0.5 (R Foundation for Statistical Computing, Vienna, Austria), to generate predictive maps of odds ratios (ORs) of HIV infection over study areas and for other risk factors (34, 35). GAMs are the extensions of traditional linear regression models, like logistic regression models for binary data, that include smooth terms to allow for the relaxation of typical linearity assumptions between predictors and outcomes (32). The model provides a unified statistical framework that allows both parametric and non-parametric bivariate smooth term of geolocation parameters (longitude and latitude) to model the spatial effects of the geographical locations, while simultaneously adjusting for covariates.

For this study, we define *y*_*ij*_ = 1 if a respondent in province *i* tested positive for HIV and *y*_*ij*_ = 0 otherwise. *y*_*ij*_ follows a Bernoulli distribution with *P*(*y*_*ij*_ = 1) = π_*ij*_ being the probability that the HIV status of the *jth* respondent in the *ith* geographical location is positive and *P*(*y*_*ij*_ = 0) = 1−π_*ij*_ is the probability that the HIV status of the *jth* respondent in the *ith* geographical location is negative. Then, the GAM model is given by:


(1) $$\begin{array}{l} logit\left(\pi_{ij}\right)=\eta_{ij}={Z^{\prime }}_{ij}\beta +S\left(x_{i},\nu_{i}\right) \end{array}$$

where logit(π_*ij*_) is the logit link function, η_*ij*_ is an additive predictor for the *jth* respondent of province *i*, β is a vector of unknown fixed regression parameters corresponding to the set of covariates (*Z*_*ij*_), *S*(*x*_*i*_, ν_*i*_) is a 2-dimensional non-parametric smooth function that is used to model geolocation of respondents for the spatial analysis, *x*_*i*_ and ν_*i*_ are the two geolocation parameters (longitude and latitude).

The main goal of the spatial analysis was to evaluate if geolocation was associated with HIV prevalence in South Africa. This study used a two-dimensional locally weighted regression smoother (LOESS) to smooth over the latitude and longitude of the individual geographical locations (33, 36). The LOESS utilized a dataset from adjacent data points to predict the odds of HIV across South Africa. Subsequently, a regular prediction grid of points was created within the study areas and a *modgam* function in the MapGAM package was used to fit the generalized additive models based on the SADHS data and to create spatial predictions on the defined grid points. The *modgam* function in the MapGAM package was used to fit the GAMs and to estimate the log-odds at the grid points and the odds ratios were estimated by the odds at every grid point using the whole study regions as reference (28, 34). The LOESS required a choice of span (i.e., tradeoff between bias and variability), and an optimal span size (% of data weighted as a distance function) for our model was determined by testing different span sizes which minimized the AIC (32, 37). Crude and covariate-adjusted spatial effects were mapped using the MapGAM package in R software. In the crude analysis, only the smooth for the two-dimensional predictor (longitude and latitude) was included in the model, while the adjusted model included all covariates in the data set. The list of covariates that were added to the adjusted model were tested for possible confounding, variables were added in the GAMs model one at a time to prevent possible confounding and the covariate-adjusted maps were obtained. We conducted a permutation test of the null hypothesis that HIV prevalence was not associated with the geographical location of the respondents, while adjusting for other risk factors (34, 38). We obtained a statistically significant *P* value of < 0.05 for testing the global spatial effect in the adjusted model.

## Results

Tables 1, 2 presents the descriptive analysis of HIV prevalence among South African women and men aged ≥15 years based on sociodemographic, geographic, biological, and behavioral characteristics of the participants.

**Table 1:** Distribution of sociodemographic, and geographic characteristics in the HIV-positive and HIV-negative South African men and women aged ≥ 15 years, SDHS data.

**Characteristics**	**Women**		**Men**	
	**HIV+ (%)**	**HIV– (%)**	**HIV+ (%)**	**HIV– (%)**
Age (Years)				
15–24	97 (11.7)	735 (88.3)	32 (3.5)	892 (96.5)
25–34	293 (36.0)	521 (64.0)	110 (17.8)	508 (82.2)
35–44	241 (40.3)	357 (59.7)	130 (26.7)	357 (73.3)
>44	49 (20.2)	194 (79.8)	96 (18.8)	415 (81.2)
Province				
Western Cape	45 (18.1)	203 (81.9)	38 (14.1)	226 (85.6)
Eastern Cape	80 (30.3)	184 (69.7)	24 (8.3)	265 (91.7)
Northern Cape	7 (14.0)	43 (86.0)	5 (10.0)	45 (90.0)
Free State	35 (28.5)	88 (86.0)	23 (18.4)	102 (81.6)
Kwazulu-Natal	182 (37.4)	305 (62.6)	79 (19.4)	328 (80.6)
Northwest	48 (29.6)	114 (70.4)	30 (16.6)	151 (83.4)
Gauteng	175 (25.4)	515 (74.6)	116 (14.9)	664 (85.1)
Mpumalanga	72 (34.1)	139 (65.9)	37 (17.4)	176 (82.6)
Limpopo	36 (14.4)	214 (85.6)	14 (6.1)	215 (93.9)
Place of Residence				
Urban	456 (27.4)	1,211 (72.6)	264 (15.1)	1,487 (84.9)
Rural	223 (27.3)	595 (72.7)	103 (13.1)	685 (86.9)
Race				
Back African	667 (29.9)	1,562 (70.1)	359 (16.3)	1,837 (83.7)
Colored	10 (6.0)	158 (94.0)	5 (2.7)	178 (97.3)
White	0 (0.0)	61 (100.0)	3 (2.4)	123 (97.3)
Indian/Asian	2 (7.7)	24 (92.3)	0 (0.0)	33 (100.0)
Educational level				
No education	18 (37.5)	30 (62.5)	14 (16.3)	72 (83.7)
Primary	93 (36.3)	163 (63.7)	71 (17.6)	332 (82.4)
Secondary	532 (27.5)	1,401 (72.5)	257 (14.5)	1,520 (85.5)
Higher	36 (14.5)	213 (85.5)	24 (8.9)	247 (91.1)
Wealth index				
Poorest	171 (30.4)	392 (69.6)	96 (18.8)	416 (81.3)
Poor	154 (32.2)	324 (67.8)	82 (16.4)	419 (83.6)
Middle	164 (29.0)	401 (71.0)	85 (16.1)	444 (83.9)
Richer	130 (26.9)	354 (73.1)	58 (10.1)	492 (89.5)
Richest	60 (15.2)	336 (84.8)	45 (10.1)	401 (89.9)
Employment status				
Unemployed	433 (25.8)	1,246 (74.2)	196 (13.4)	1,265 (86.6)
Employed	246 (30.5)	560 (69.5)	171 (15.9)	907 (84.1)
Marital status				
Never married	407 (27.0)	1,101 (73.0)	139 (9.2)	1,369 (90.8)
Married	105 (19.3)	440 (80.7)	84 (14.2)	508 (85.8)
Windowed	14 (31.8)	30 (68.2)	16 (51.6)	15 (48.4)
Divorced	6 (24.0)	19 (76.0)	5 (15.6)	27 (84.4)
Living with partner	122 (40.8)	177 (59.2)	89 (32.0)	189 (68.0)
Separated	25 (39.1)	39 (60.9)	33 (34.4)	63 (65.6)

**Table 2:** Distribution of biological and behavioral characteristics in the HIV-Positive and HIV-negative South African men and women aged ≥ 15 years from the SDHS data.

**Characteristics**	**Women**		**Men**	
	**HIV+**	**HIV-**	**HIV+**	**HIV-**
Recent sexual activities				
Never had sex	12 (4.9)	235 (95.1)	15 (5.1)	279 (94.9)
Active in last 4 weeks	377 (30.3)	866 (69.7)	226 (15.5)	1,231 (84.5)
Not active in the last 4 weeks (Not postpartum)	248 (29.3)	599 (70.7)	126 (16.0)	662 (84.0)
Number of unions				
Once	247 (26.7)	679 (73.3)	199 (22.3)	692 (77.7)
More than once	26 (50.0)	26 (50.0)	22 (17.2)	106 (82.8)
Had STI in last 12 months				
No	631 (26.6)	1,740 (73.4)	346 (14.1)	2,105 (85.9)
Yes	48 (42.5)	65 (57.5)	21 (23.9)	67 (76.1)
Ever tested for HIV				
No	56 (15.0)	318 (85.0)	56 (8.0)	643 (92.0)
Yes	623 (29.5)	1,488 (70.5)	311 (16.9)	1,529 (83.1)
Male circumcision				
No	NA	NA	207 (18.4)	916 (81.6)
Yes	NA	NA	159 (11.3)	1,251 (88.7)
Don't know	NA	NA	1 (16.7)	5 (83.3)
Had genital discharge				
No	601 (26.2)	1,689 (73.8)	349 (14.4)	2,080 (85.6)
Yes	68 (36.8)	117 (63.2)	15 (15.8)	80 (84.2)
Don't know	9 (90.0)	1 (10.0)	3 (21.4)	11 (78.6)
Had genital ulcer/sore				
No	617 (25.9)	1,765 (74.1)	349 (14.2)	2,112 (85.8)
Yes	53 (55.8)	42 (44.2)	15 (22.4)	52 (77.6)
Don't know	9 (100.0)	0 (0.0)	3 (27.3)	8 (72.7)
Use of contraceptives and method type				
Never used	325 (24.9)	982 (75.1)	169 (13.9)	1,046 (86.1)
Used traditional/folkloric method	1 (20.0)	4 (80.4)	1 (50.0)	1 (50.0)
Used modern methods	352 (30.0)	821 (70.0)	197 (14.9)	1,124 (85.1)
Frequency of reading newspaper or magazine				
Not at all	295 (32.4)	616 (67.6)	113 (14.5)	668 (85.5)
Less than once a week	182 (28.9)	447 (71.1)	94 (11.8)	700 (88.2)
At least once a week	202 (21.4)	743 (78.6)	159 (16.5)	804 (83.5)
Frequency of watching television				
Not at all	144 (29.0)	352 (71.0)	54 (16.5)	274 (83.5)
Less than once a week	61 (29.2)	148 (70.8)	56 (14.1)	342 (85.9)
At least once a week	474 (26.6)	1,306 (73.4)	257 (14.2)	1,556 (85.8)
Frequency of using the internet				
Not at all	467 (33.5)	926 (66.5)	252 (19.4)	1,045 (80.6)
Less than once a week	19 (20.0)	76 (80.0)	10 (8.9)	102 (91.1)
At least once a week	56 (23.0)	188 (77.0)	39 (11.7)	294 (88.3)
Almost every day	137 (18.2)	616 (81.8)	66 (8.3)	730 (91.7)

Results of the adjusted odds ratios (AORs) and their corresponding 95% confidence intervals (CI) using GAMs with spatial effects were displayed in Tables 3, 4. From the GAMs analyses, we found that HIV infection was negatively associated with socioeconomic status. The odds of HIV infection were statistically significantly lower in both women (AOR 0.20, 95% CI 0.11, 0.36) and men (AOR 0.21, 95% CI 0.20, 0.40) with highest wealth quintile (richest) as compared to their poorest counterparts. Similarly, the study found that higher education attainment has a protective effect against HIV infection among men and women. The odds of HIV infection were lower among men (AOR 0.35, 95% CI 0.12, 0.98) and women (AOR 0.43, 95% CI 0.21, 0.88) who attained highest educational level as compared to their counterparts who did not go to school, and those who attained only primary and secondary education, although these differences were not significant. Significantly lower odds of HIV were found among employed men (AOR 0.68, 95% CI 0.47, 0.97) and women (AOR 0.79, 95% CI 0.62, 0.98) as compared to their counterparts who were not employed. The odds of HIV infection increased significantly among women aged 25–34 years (AOR 1.86, 95% CI 1.10, 3.13), and 34–44 years (AOR 1.85, 95% CI 1.09, 3.12). Similarly, the odds of HIV infection among men who are between 34 and 44 years were 5.61 times higher (AOR 5.61, 95% CI 1.29, 8.48) compared to their younger counterparts. Surprisingly, the odds of HIV infection among men residing in rural areas were 1.60 times higher (AOR 1.60, 95% CI 1.12, 2.29) as compared to those residing in urban areas. Similarly, the odds of HIV infection among women were 1.65 times higher (AOR 1.65, 95% CI 1.28, 2.12) as compared to their counterparts in urban areas. Widowed/divorced/separated men (AOR 1.82, 95% CI 1.18, 2.80) and women (AOR 2.31, 95% CI 1.62, 3.27) had statistically significant higher odds of HIV as compared to their counterparts.

**Table 3:** Adjusted GAMs analysis results: Adjusted odds of HIV-positive among South African men and women aged ≥ 15 years who tested for HIV during the SADHS, according to selected sociodemographic, geographic, and behavioral.

**Characteristics**	**Women**		***P*-value**	**Men**		***P*-value**
	**AOR**	**95% CI**		**AOR**	**95%CI**	
Age						
15–24 years	Ref.	Ref.		Ref.	Ref.	
25–34 years	1.86	[1.10, 3.13]	**0.045**	3.31	[0.75, 9.68]	0.114
35–44 years	1.85	[1.09, 3.12]	**0.048**	5.61	[1.29, 8.48]	**0.021**
>44 years	1.29	[0.72, 2.29]	1.431	3.16	[0.73, 7.77]	0.125
Place of Residence						
Urban	Ref.	Ref.		Ref.	Ref.	
Rural	1.65	[1.28, 2.12]	**0.018**	1.60	[1.12, 2.29]	**0.010**
Race						
Black Africa	Ref.	Ref.		Ref.	Ref.	
White/colored	0.26	[0.12, 0.53]	**< 0.001**	0.08	[0.05, 0.29]	**< 0.001**
Indian/Asian	0.19	[0.02, 1.57]	0.122	0.02	[0.01, 0.14]	**< 0.001**
Educational level						
No education	Ref.	Ref.		Ref.	Ref.	
Primary	1.28	[0.68, 2.37]	1.639	2.02	[0.97, 4.20]	0.060
Secondary	0.94	[0.54, 1.67]	3.032	1.45	[0.73, 2.88]	0.286
Higher	0.43	[0.21, 0.88]	**0.031**	0.35	[0.12, 0.98]	**0.040**
Wealth index						
Poorest	Ref.	Ref.		Ref.	Ref.	
Poor	1.06	[0.75, 1.49]	2.669	0.68	[0.45, 1.11]	0.454
Middle	0.98	[0.69, 1.37]	3.252	0.63	[0.38, 1.03]	0.879
Richer	0.79	[0.54, 1.15]	0.818	0.31	[0.17, 0.55]	**0.045**
Richest	0.20	[0.11, 0.36]	**0.001**	0.21	[0.20, 0.40]	**0.015**
Employment status						
Unemployed	Ref.	Ref.		Ref.	Ref.	
Employed	0.79	[0.62, 0.98]	**0.048**	0.68	[0.47, 0.97]	**0.032**
Marital status						
Never in union	Ref.	Ref.		Ref.	Ref.	
Married/Living with partner	2.37	[1.73, 3.25]	0.340	0.42	[0.54, 1.23]	0.541
Windowed/divorced/separated	2.31	[1.62, 3.27]	**0.047**	1.82	[1.18, 2.80]	**0.012**
Number of unions						
Once	Ref.	Ref.		Ref.	Ref.	
More than once	2.10	[1.42, 3.10]	**0.034**	1.09	[0.64, 1.89]	0.730

Ref, Reference; AOR, Adjusted Odds ratio; CI, Confidence.

*P < 0.05 was considered statistically significant at a 95% confidence level.

Bold values indicate all P-values that are < 0.05 and statistically significant.

**Table 4:** Adjusted GAMs results: Adjusted odds of HIV-positive among South African men and women aged ≥ 15 years who tested for HIV during the SADHS, according to selected behavioral and biological covariates.

**Characteristics**	**Women**		**P-value**	**Men**		***P*-value**
	**AOR**	**95% CI**		**AOR**	**95%CI**	
Recent sexual activities						
Never had sex	Ref.	Ref.		Ref	Ref.	
Active in last 4 weeks	0.84	[0.41, 1.72]	0.630	0.72	[0.35, 1.32]	0.271
Not active in the last 4 weeks	0.89	[0.43, 1.88]	0.771	0.64	[0.54, 1.51]	0.306
Had STI in the last 12 months						
No	Ref.	Ref.		Ref.	Ref.	
Yes	1.26	[0.77, 2.04]	1.312	1.76	[1.44, 3.68]	**0.035**
Ever tested for HIV						
No	Ref.	Ref.		Ref.	Ref.	
Yes	1.32	[0.75, 2.34]	0.342	1.24	[0.76, 2.02]	0.384
Male circumcised						
No	Ref.	Ref.		Ref.	Ref.	
Yes	NA	NA		0.73	[0.52, 0.98]	**0.042**
Had genital discharge						
No	Ref.	Ref.		Ref.	Ref.	
Yes	1.73	[1.62, 3.29]	**0.040**	1.25	[0.48, 3.28]	0.638
Had genital ulcer/sore						
No	Ref.	Ref.		Ref.	Ref.	
Yes	1.90	[1.22, 2.96]	**0.049**	2.25	[1.91, 5.59]	**0.050**
Frequency of using the internet						
Not at all						
Less than once a week	0.79	[0.41, 1.54]	1.804	0.82	[0.32, 2.09]	0.669
At least once a week	0.57	[0.34, 0.96]	0.454	0.62	[0.32, 1.21]	0.159
Almost every day	0.39	[0.27, 0.55]	**0.001**	0.40	[0.24, 0.67]	**< 0.001**
Frequency of reading newspaper or magazine						
Not at all	Ref.	Ref.		Ref.	Ref.	
Less than once a week	0.75	[0.56, 1.02]	0.942	0.83	[0.58, 1.29]	0.423
At least once a week	0.59	[0.44, 0.78]	**0.048**	0.76	[0.54, 0.87]	**0.051**
Frequency of watching television						
Not at all	Ref.	Ref.		Ref.	Ref.	
Less than once a week	0.71	[0.43, 1.17]	0.650	1.41	[0.76, 2.61]	0.273
At least once a week	0.18	[0.62, 1.12]	0.826	0.89	[0.55, 1.48]	0.671

Ref, Reference; AOR, Adjusted Odds ratio; CI, Confidence.

*P < 0.05 was considered statistically significant at a 95% confidence level.

Bold values indicate all P-values that are < 0.05 and statistically significant.

Furthermore, this study observed that male circumcision was statistically significantly associated with HIV infection. Men who were circumcised had lower odds of HIV infection (AOR 0.73, 95% CI 0.52, 0.98) compared to their counterparts who were not circumcised. Men who had an STIs in the last 12 months prior to the survey were found to have higher odds of HIV infection (AOR 1.76, 95% CI 1.44, 3.68) as compared to their counterpart. Contrarily, we obtained no significant association between STIs in women and HIV infection. The odds of HIV infection were 2.25 times higher among men who had prior genital ulcer/sore (AOR 2.25, 95% CI 1.91, 5.59), and 1.90 times higher among women who had prior genital ulcer/sore (AOR 1.90, 95% CI 1.22, 2.96) as compared to those who had no genital sore. The odds of HIV infection were 1.73 times higher for women who had prior genital discharge (AOR 1.73, 95% CI 1.62, 3.29) compared to those women with no history of genital discharge, although no significant association was obtained for men. The length of marriage and number of unions have no significant relationship with HIV risks for both genders. However, being sexually active increased the odds of HIV in women as those who had multiple sexual partners had higher odds of HIV infection (AOR 2.10, 95% CI 1.42, 3.10). There was a strong significant negative effect of mass media on HIV prevalence, the study found that those men (AOR 0.40, 95% CI 0.24, 0.67) and women (AOR 0.39, 95% CI 0.27, 0.55) who use the internet almost every day had lower odds of HIV infection as compared to their counterparts. Similarly, men (AOR 0.76, 95% CI 0.54, 0.87) and women (AOR 0.59, 95% CI 0.44, 0.78) who read newspapers at least once a week had lower odds of HIV infection as compared to their counterparts. However, no significant association between those who watch television and HIV infection was observed.

### Results of spatial analysis

The GAM model was employed for the spatial analysis to identify clusters of the HIV outcome (spatial regions with statistically significant HIV high or low risks). A global spatial effect test based on deviance statistic (34, 37), was also carried out to determine whether geolocation was significant in the study and we obtained a *P* value = 0.001 for all analyses, results suggest that the prevalence of HIV was significantly linked with the geo-location of the respondents. The optimal scan size which minimized the Akaike Information Criterion (AIC) was found to be 0.1, indicating that 10% of the adjacent dataset were used for smoothing the geo-location parameters (latitude and longitude). We calculated the 95% confidence intervals for point estimates of the HIV risk map and areas with confidence intervals excluding one are shown on the map with contour black lines and the absence of such line indicates no statistically significant odd ratios (34).

**Figure 1:**
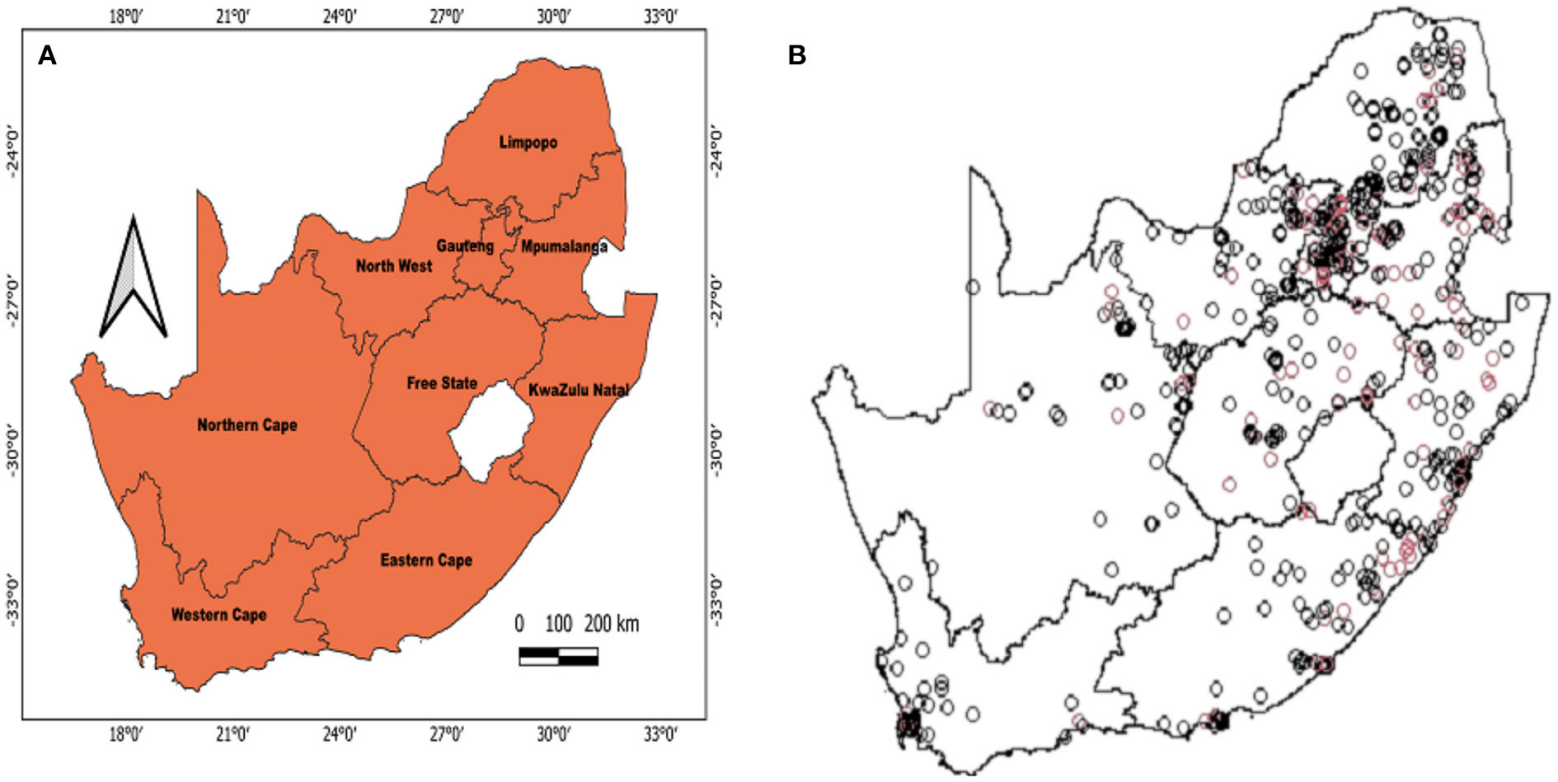
Map of South Africa that shows where the survey dataset was collected based on the SDHS-16 **(A)** and the geolocations of HIV observations **(B)**. Depicted in **(B)** are HIV+ cases (red) and HIV- cases (black).

**Figure 2:**
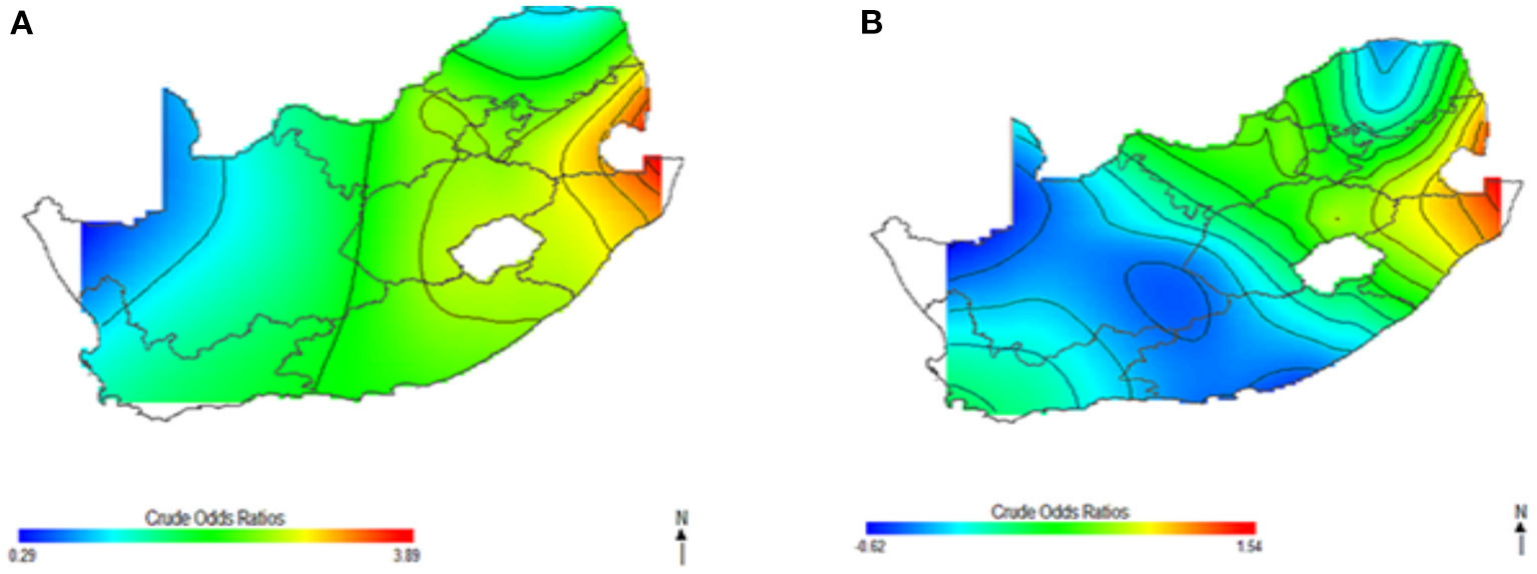
Map of crude odds ratios for women **(A)** and men **(B)** using optimal span size of 0.50. Black contour bands indicate statistically significant areas of increased or decreased HIV risks.

Figure 1 shows the geolocation of HIV observations across the study areas. The study mapped both crude and covariate-adjusted spatial effects to describe the relationship of HIV risk to spatial locations. The geographical distribution of unadjusted (crude) HIV odds ratios in Figures 2A,B shows creased risks of HIV in east coast, central and north-eastern regions, with the highest odds ratios (ORs) at 3.9 (global *p*-value < 0.01; span size = 0.5). Conversely, respondents living in west coast and northern regions had significantly lower odds of HIV. The local test detected regions of statistically and significantly higher and lower HIV risks which are signified by black lines in Figure 2. The crude map has indicated a spatial pattern of HIV that agreed with the geographical distribution of respondents with HIV positive outcomes.

**Figure 3:**
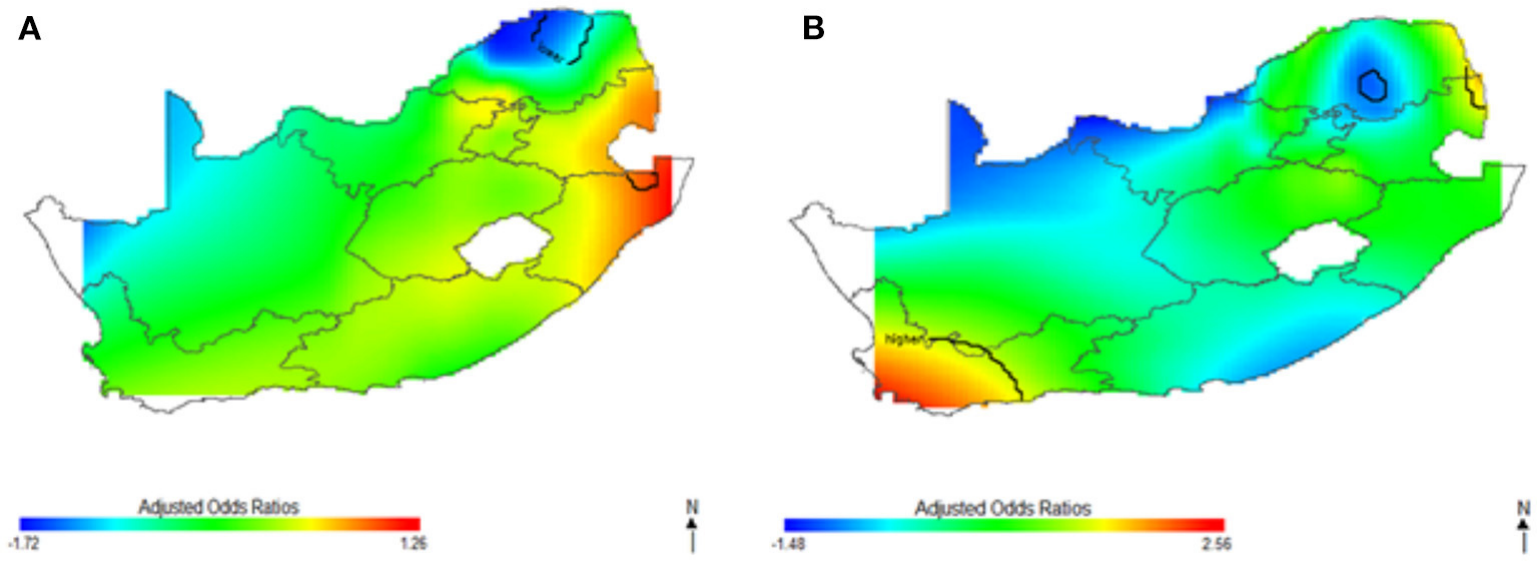
Map of adjusted odds ratios for women **(A)** and men **(B)** showing spatial distribution of HIV risks across South African provinces, adjustment for sociodemographic, biological, and behavioral covariates using optimal span size of 0.70. Back contour bands indicate significant areas of increased or decreased HIV odds.

Figure 3 shows the spatial distribution of the covariate-adjusted HIV odds ratios for South African women aged 15–49 year (Figure 3A) and men aged 15–49 years (Figure 3B). The *p*-value for testing the global spatial effects of HIV was 0.0004, and result suggested a significant association between geolocation and HIV prevalence after adjusting for socioeconomic, demographic, and behavioral factor. The spatial patterns of adjusted HIV odds ratio across South African regions showed significant variation of HIV risks. Adjusting for covariates decreased the size of geographical areas of higher and lower HIV odds for men in Figure 3B; although, study's results continued to indicate spatial variability in the odds of HIV, especially in northeast coast region. The study still observed a significant relationship between geolocation and HIV prevalence after covariate adjustment with global *p*-value of < 0.01. Most geographical regions showed significantly changed the odds ratios, and statistically significantly increased odds of HIV were observed among women (A) and men (B) residing in east coast, central and north-eastern regions. However, respondents in west coast and northern regions showed statistically significant decreased odds of HIV. We also observed in Figure 3B, a possible shift of increased HIV odds ratios to southwest coast region, indicating possibility of local spatial effects unobserved by the adjusted covariates. Notably, HIV risk remained elevated in northeast coast region, while southwest coast region had significantly increased odds ratios for men after covariate adjustment (Figure 3B).

**Figure 4:**
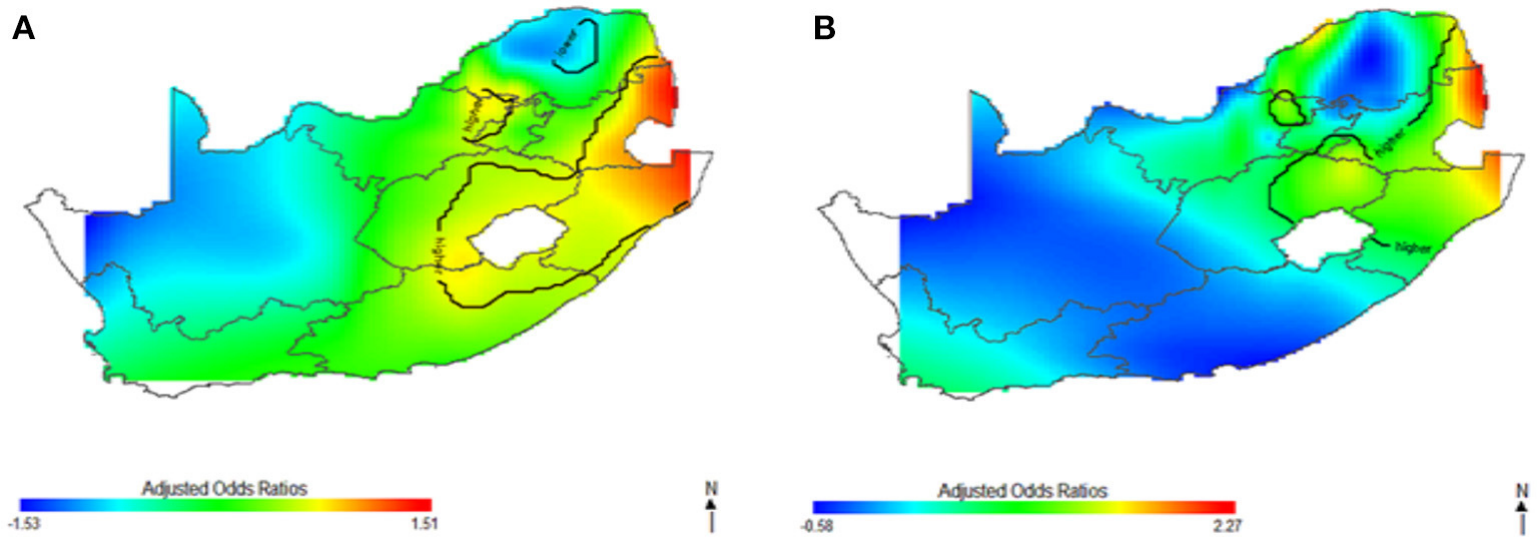
The contribution of behavioral factors on the spatial distribution of HIV odds among women **(A)** and men **(B)**. The black contour lines indicate areas where the upper band and lower band exclude one.

This study captured potential differences in the odds of HIV separately and to identify the contributions of strong sociodemographic predictors on HIV risks. Figure 4 shows the contribution of age, wealth index, type of place of residence, educational status, and employment status to spatial patterns of HIV odds ratios associated with respondent's geolocation. We observed fluctuation of increased odds of HIV among women (Figure 4A) and men (Figure 4B) in relation to each factor, however, we still observed highest odds of HIV in northeast coast region. Respondents living in west coast and northern regions showed significantly lower odds of HIV as regards the summary contribution of each variable. It is clear in Figure 4 that the unadjusted maps have similar patterns, and the spatial disparities in HIV risk remained even after adjustment of covariates. The results suggest that the identified confounding factors are not the only influential factors to the spatial patterns of HIV risks in South Africa.

**Figure 5:**
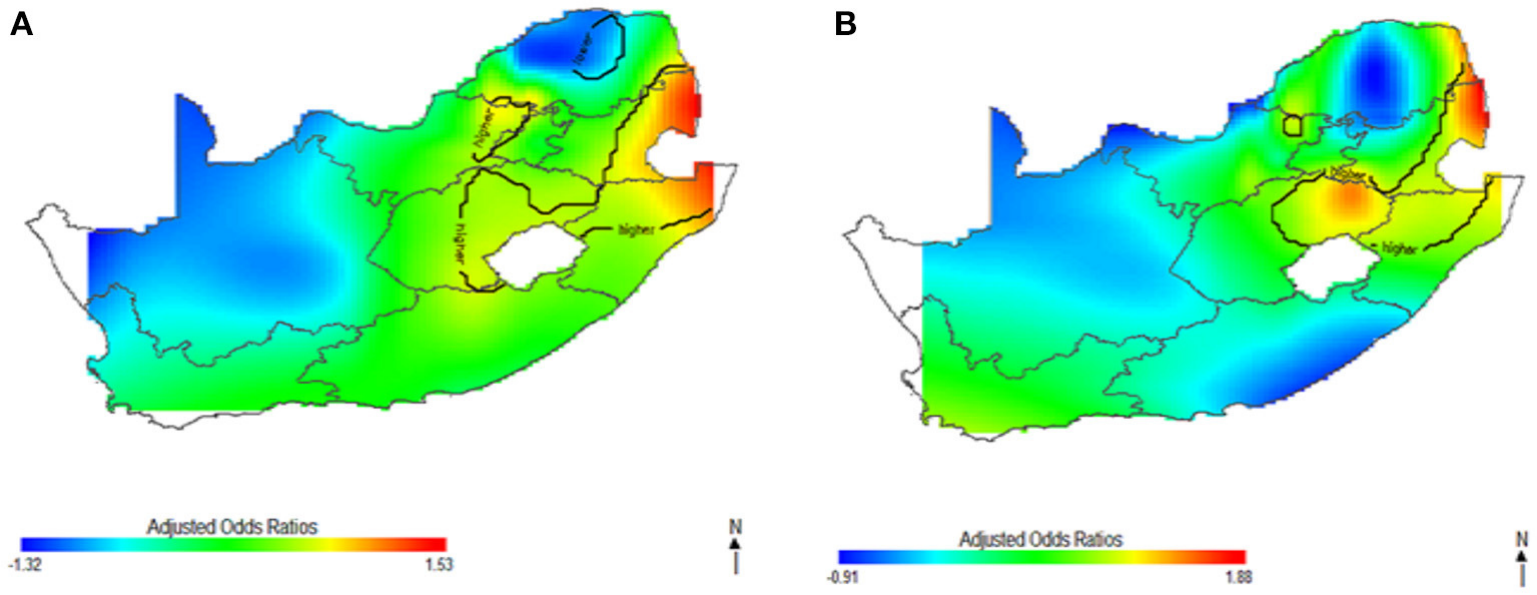
The contribution of sociodemographic factors on the spatial distribution of HIV odds among women **(A)** and men **(B)**. The black contour lines indicate areas where the upper band and lower band exclude one.

**Figure 6:**
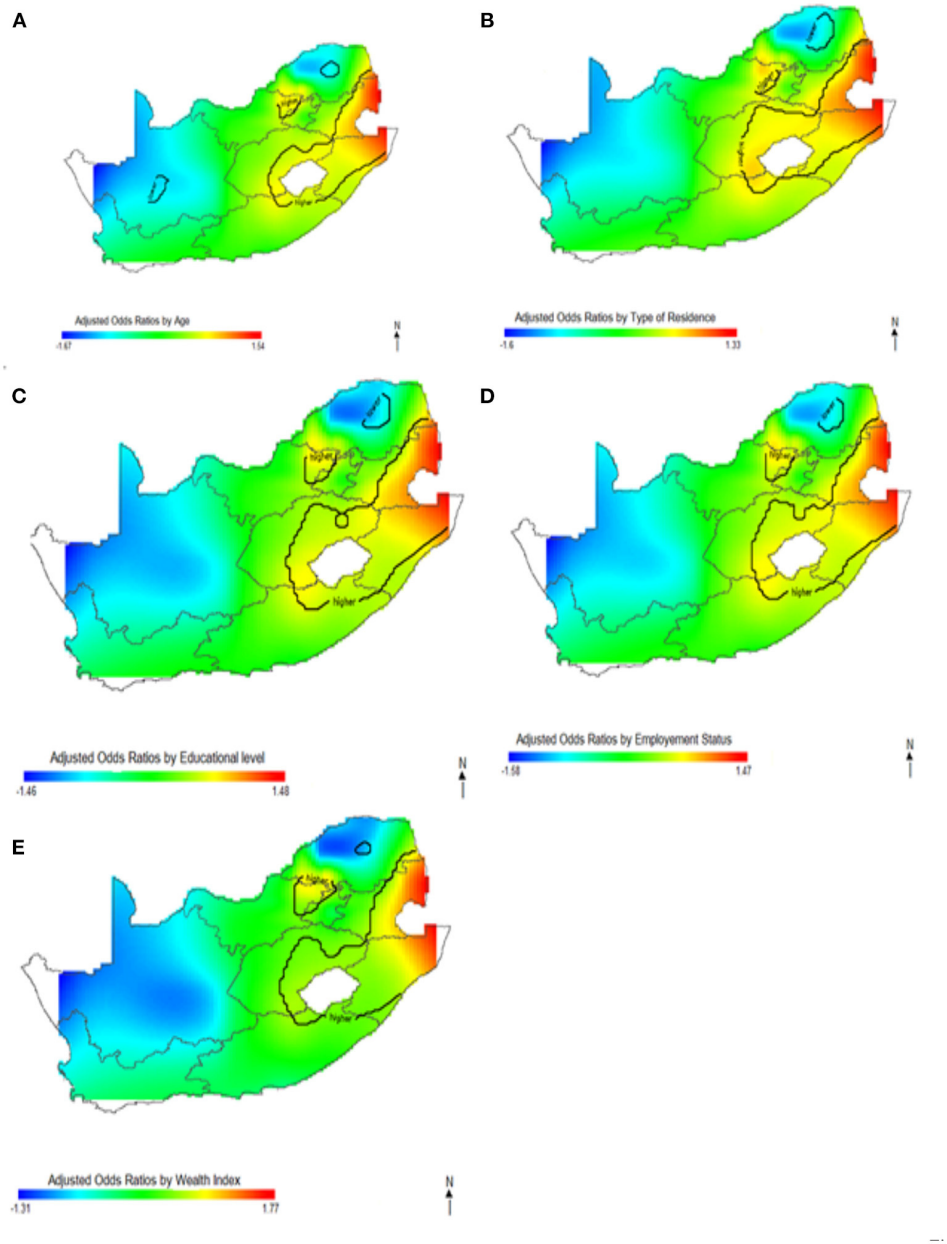
Contributions of potential risk factors such as age **(A)**, place of residence **(B)**, educational level **(C)**, employment status **(D)** and wealth index **(E)** to the spatial distribution of HIV prevalence for women. The black contour lines indicate areas where the upper band and lower band exclude one.

**Figure 7:**
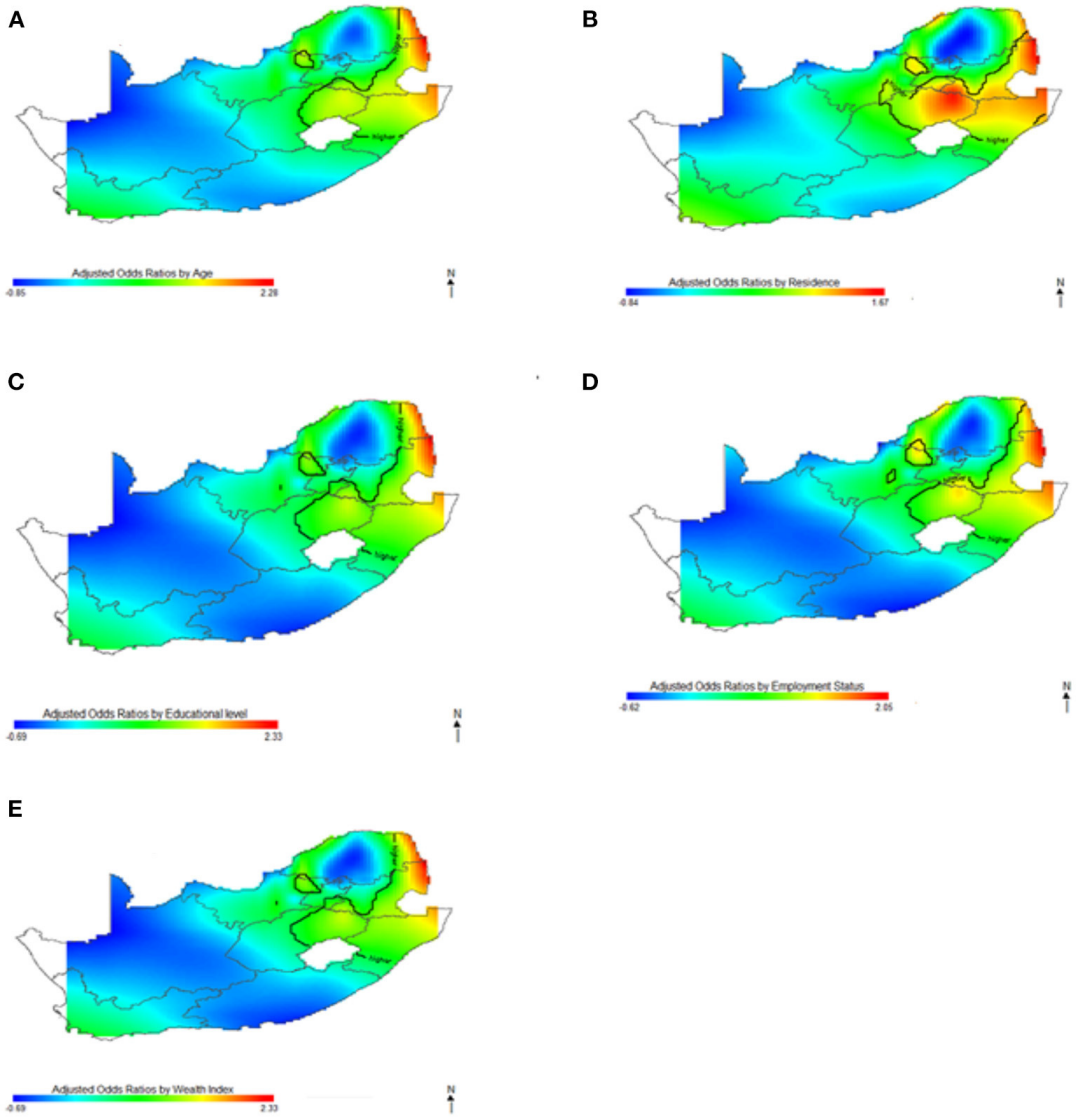
Contributions of potential risk factors such as age **(A)**, place of residence **(B)**, educational level **(C)**, employment status **(D)** and wealth index **(E)** to the spatial distribution of HIV prevalence for men. The black contour lines indicate areas where the upper band and lower band exclude one.

In Figures 5–7, the maps show the contributions of each potential risk factors to the geographic patterns of HIV odds ratios associated with women (Figure 6) and men (Figure 7), after controlling for other effects. The Figures show how important factors such as age, type of residence, educational level, employment status and wealth index have varying effect on HIV prevalence across geographical regions when compared with their references. Whereas these factors have positive effect in some regions, the effects are negative in other regions. For instance, we observed in Figure 6 women residing in east coast, central and north-eastern regions who had no education, younger, resided in rural areas, poorer and had no employment had higher odds of HIV as compared to their counterpart in northern regions. Similarly, in Figure 7 we observed that men who resided in the same east coast, central and north-eastern regions who had no education, poorer, no employment, and resided in rural areas had higher odds of HIV as compared to their counterparts in other regions.

## Discussion

The findings revealed differences in spatial variation of HIV prevalence based on the respondent's location and gender, which were explained substantially by sociodemographic, behavioral, and biological factors. Changes in the location and magnitude of significance of HIV odds were observed in both crude and adjusted analyses, but gender differences in HIV spatial variation were observed after adjusting for sociodemographic, behavioral, and biological factors. The observed spatial disparity pattern for women remained constant, whereas we discovered a significant shift in HIV spatial patterns for men.

This study found significantly higher odds of HIV infection among both men and women in east coast, central and north–eastern regions based on the results of our crude analysis. Surprisingly after the adjustment of socioeconomic, demographic, behavioral and biological factors in the adjusted spatial analyses, we discovered a significant shift in HIV spatial patterns for men. The significant area of highest odds of HIV for the men shifted from south coast region to northeast coast region with little evidence of variation. However, spatial variation of HIV odds for women increased in magnitude, with elevated HIV burden in east coast, central and north-eastern regions. Consistently, northeast coast region have remained low risk area with and without covariate adjustment, contrary to the findings of Kleinschmidt et al. (39). The results may suggest that risk factors related to a particular area (local factors) influenced the increased odds of HIV for men. The risk of HIV expanded to other provinces for women after covariate adjustment, and there was small-scale variation of HIV odds among the men. Similarly, the lower risk of HIV expanded to other provinces for men (northwestern and southeast coast) but did not change substantially for women. The observed increased odds of HIV infection in northeast coast region remained significantly elevated even after covariate adjustment.

Among women and men, differences in the odds of HIV infection by place of residence varied widely by province, from the lowest odd in northern region to the highest odd in northeast coast region. It was evident from our analyses that northeast coast region is the highest risk area for HIV associated with both genders, but only the males experienced elevated odds of HIV in northeast coast region. The observed gender differences in the spatial distribution of HIV are of public health concern. Often, spatial analysis focuses on high-risk regions, for condition like HIV. However, low risk regions (decreased odds) may be of greater public health concern, that may suggest possibility that HIV is undiagnosed in these areas. As high regions are identified, assessing area with low HIV prevalence may be helpful in recognizing regions in critical need of health-care services.

Notably, several studies have been published in the South African HIV epidemiological literature assessing the geographical disparities in the prevalence of risk factors and HIV disease outcomes. Tanser et al. (40), utilized Gaussian kernel analysis to model population-based household and HIV surveillance data in South Africa, and the findings of the study have revealed considerable geographical variation in local HIV prevalence, indicating the need for interventions that target the highest HIV-risk areas in South Africa to supplement measures aimed at the general population. Using data from one of the most comprehensive demographic surveillance systems in South Africa, Diego et al. (41) mapped HIV prevalence and discovered that over 60% of HIV transmission was directly linked to a geographical cluster in peri-urban areas. Their study confirmed the findings of our study, which indicated a high level of connectivity between HIV geographical high-risk populations and the northeast region as a whole. Handan et al. (42) examined spatial disparities in high-risk women and HIV infections in South Africa using generalized additive models (GAMs). The GAM model was fitted to decade-long data (2002–2012) from northeast region, and the study's findings revealed significant spatial clustering of HIV in areas with the highest risk of infection. Similarly, a recent study by Chimoyi et al. (43) evaluated the spatial patterns of HIV prevalence and interventions in semi-urban settings in South Africa, and their findings indicated clusters with high HIV infection across urban and semi-urban districts in northcentral region, South Africa, indicating the need to strengthen other HIV prevention programmes in high-risk areas. These studies confirmed the existence of spatial heterogeneity in the prevalence of HIV in South Africa, however, different from their spatial analyses, the use of MapGAM in our present study with a non-parametric bivariate smooth term of geolocation parameters provided a unified framework for our spatial prediction, estimation, and inference on covariate-adjusted spatial effects using individual-level data. In addition, based on our gender-specific analyses, we discovered that the prevalence of HIV among men and women in various regions varies greatly.

The results of the varying effects of sociodemographic, behavioral, and biological variables examined in this study displayed substantial effect and are intriguing. This study has observed differences in the spatial variation of HIV for men and women as explained by these important variables. We found that higher HIV prevalence for women is mainly concentrated in east coast, central and north-eastern regions as explained by these variables, while for men, HIV varied spatially across northeast coast, central and southwest regions. The odds of HIV infection were significantly higher in both men and women between the ages of 35 and 44 years. Surprisingly, the study found that women between the ages of 25–34 years had statistically higher odds of HIV infection, but no significant association was found for men in the same age group. This suggests that the HIV epidemic in South Africa primarily affects younger to middle-aged men and women, and the risk reduces as age increases. These findings are consistent with other results from researchers in sub-Saharan Africa (15, 44). The gender disparity in HIV prevalence emphasizes the potential benefit of gender-age-specific HIV interventions and highlights the need for targeted programs that could interrupt HIV transmission especially in young women aged between 25–44 years.

Our study had some strengths worth mentioning. In this study, we used GAMs to identify nation-wide spatial disparities of HIV prevalence in South Africa. GAMs allow for identification of spatial disparities while systematically determining predictors of the spatial disparities. Identification of spatial disparities of HIV prevalence and where spatial disparities persist after adjustment for factors may provide additional insight into unexplored factors that vary by location. Furthermore, we conducted a sex-stratified analyses, to provide additional insight into sex-specific factors. Using SADHS is a nationally representative sample with a using geocoded data, allowed mapping effect estimates of key factors that could guide geographically targeted intervention. Despite the methodological strengths, our study had some potential limitations. The study was limited to the SADHS measured variables and important data on migration patterns, key populations, coverage of anti-retroviral therapy (ART) and other prevention interventions were not included in our analysis (30). The cross-sectional nature of SADHS data precluded examination of the causal relationship between HIV prevalence and observed risk factors.

## Conclusion

HIV prevalence is not spatially uniform across South Africa and spatially varied depending on gender. Our study identified areas with statistically significant increased and decreased HIV risk by using GAMs to smooth the effect of geographical locations. By stratifying our analyses by gender, we were able to identify significant geographical disparities in HIV prevalence by location between men and women, providing evidence that gender influences HIV vulnerability and exposure. This study found that men and women living in rural areas were more likely to be HIV-positive than their urban counterparts. Intervention programs aimed at reducing the HIV burden in South Africa by 2030 must focus on HIV awareness, control, and prevention programs aimed at vulnerable non-educated, unemployed, and poor people living in rural communities across South Africa, particularly in KwaZulu-Natal. Further research into the complex interaction between geolocation and other HIV risk factors in South Africa that were not considered in this study may be required.

## Data availability statement

Publicly available datasets were analyzed in this study. This data can be found here: https://dhsprogram.com/data/available-datasets.cfm.

## Ethics statement

Ethical review and approval was not required for the study on human participants in accordance with the local legislation and institutional requirements. Written informed consent to participate in this study was provided by the participants' legal guardian/next of kin.

## Author contributions

CU obtained permission to use the 2016 SDHS/geospatial data sets and performed the analysis. JN conceptualized the modeling idea. CU and JN jointly drafted and revised the manuscript. Both authors read and approved the final manuscript for submission.

## Conflict of interest

The authors declare that the research was conducted in the absence of any commercial or financial relationships that could be construed as a potential conflict of interest.

## Publisher's note

All claims expressed in this article are solely those of the authors and do not necessarily represent those of their affiliated organizations, or those of the publisher, the editors and the reviewers. Any product that may be evaluated in this article, or claim that may be made by its manufacturer, is not guaranteed or endorsed by the publisher.
